# Improvement of *Bacillus subtilis* for poly‐γ‐glutamic acid production by genome shuffling

**DOI:** 10.1111/1751-7915.12405

**Published:** 2016-08-26

**Authors:** Wei Zeng, Guiguang Chen, Hao Wu, Jun Wang, Yanliao Liu, Ye Guo, Zhiqun Liang

**Affiliations:** ^1^ State Key Laboratory for Conservation and Utilization of Subtropical Agro‐bioresourcesGuangxi UniversityNanningGuangxi530004China; ^2^ Key Laboratory of Ministry of Education for Microbial and Plant Genetic EngineeringGuangxi UniversityNanningGuangxi530004China; ^3^ College of Life Science and TechnologyGuangxi UniversityNanningGuangxi530004China

## Abstract

Poly‐γ‐glutamic acid (γ‐PGA) is a promising microbial polymer with potential applications in industry, agriculture and medicine. The use of high γ‐PGA‐producing strains is an effective approach to improve productivity of γ‐PGA. In this study, we developed a mutant, F3‐178, from *Bacillus subtilis* GXA‐28 using genome shuffling. The morphological characteristics of F3‐178 and GXA‐28 were not identical. Compared with GXA‐28 (18.4 ± 0.8 g l^−1^), the yield of γ‐PGA was 1.9‐fold higher in F3‐178 (34.3 ± 1.2 g l^−1^). Results from batch fermentation in 3.7 l fermenter showed that F3‐178 was satisfactory for industrial production of γ‐PGA. Metabolic studies suggested that the higher γ‐PGA yield in F3‐178 could be attributed to increased intracellular flux and uptake of extracellular glutamate. Real‐time PCR indicated that mRNA level of *pgs*B in F3‐178 was 18.8‐fold higher than in GXA‐28, suggesting the higher yield might be related to the overexpression of genes involved in γ‐PGA production. This study demonstrated that genome shuffling can be used for rapid improvement of γ‐PGA strains, and the possible mechanism for the improved phenotype was also explored at the metabolic and transcriptional levels.

## Introduction

Poly‐γ‐glutamic acid (γ‐PGA), a microbial polymer, is synthesized inside the cell via amide linkages between the α‐amino and γ‐carboxylic groups of glutamic acid residues (Shih and Van, 2001). As γ‐PGA is non‐toxic, edible, plastic, biocompatible and biodegradable, it has become a promising biomaterial with potential applications in industry, agriculture, medicine, food, cosmetics and water treatment (Bajaj and Singhal, 2011). Great efforts have been made for large‐scale production, such as strain selection, use of cheaper and renewable substrates, and optimization of fermentation process. The development of high γ‐PGA‐producing strains is one of the most effective methods to improve productivity of γ‐PGA.

The majority studies on γ‐PGA strains have been focused on wild‐type *Bacillus* species, such as *B. subtilis* IFO3335 (Goto and Kunioka, 1992), *B. subtilis* F‐2‐01 (Kubota *et al*., 1993), *B. subtilis* NX‐2 (Xu *et al*., 2005), *B. subtilis* ZJU‐7 (Shi *et al*., 2006), *B. subtilis* RKY3 (Jeong *et al*., 2014), *B. licheniformis* ATCC 9945a (Ko and Gross, 1998), *B. licheniformis* WX‐02 (Wei *et al*., 2010) and *B. amyloliquefaciens* LL3 (Cao *et al*., 2011), which produced γ‐PGA usually at the yields of more than 10 g l^−1^. However, the yield of γ‐PGA still need to be further improved. Recently, several studies have tried to apply genetic engineering of recombinant *Escherichia coli* to improve the productivity. For example, *E. coli* JM109/pPGS1 + pBSGR3 which carries γ‐PGA synthetase operon (*pgs*BCA) and glutamate racemase gene (*glr*) of *B. subtilis* IFO 3336 was constructed (Ashiuchi *et al*., 1999), but the γ‐PGA yield was only 24 mg l^−1^ which is obviously lower than that of wild‐type *Bacillus* species. The γ‐PGA concentration was 3.7 g l^−1^ in recombinant *E. coli* with *pgs*BCA of *B. subtilis* (chungkookjang) and constitutive HCE promoter (Jiang *et al*., 2006). The *B. amyloliquefaciens* LL3ΔUBG, a *gudB*/*rocG* double mutant, produced 5.68 g l^−1^ γ‐PGA compared with 4.03 g l^−1^ for the wild type, a 40% increase (Zhang *et al*., 2015). Although certain improvements have been achieved in previous investigations, the yield is still not satisfactory for industrial production of γ‐PGA.

During the past several years, an efficient technology named genome shuffling had been used to improve the industrial microbial phenotypes. It is a laboratory evolution method via recombination of multiple parents by several rounds of genome fusion. Comparing with genetic engineering, it allows genetic changes at whole genome level and does not require the genome sequence data and metabolic network information (Zhang *et al*., 2002; Biot‐Pelletier and Martin, 2014). Recent reports have described the use of genome shuffling to improve production of lipopeptide by *B. amyloliquefaciens* (Zhao *et al*., 2012), avilamycin by *Streptomyces viridochromogenes* (Lv *et al*., 2013) and transglycosylation activity by *Aspergillus niger* (Li *et al*., 2014). However, genome shuffling method has not been applied to improve the production of γ‐PGA.

In this study, we developed a strain from *B. subtilis* GXA‐28 using genome shuffling. We examined the yield of γ‐PGA and performed metabolic analysis of γ‐PGA biosynthesis. We also determined the mRNA levels of genes implicated in γ‐PGA biosynthesis.

## Results and discussion

### Strain mutagenesis

**Figure 1:**
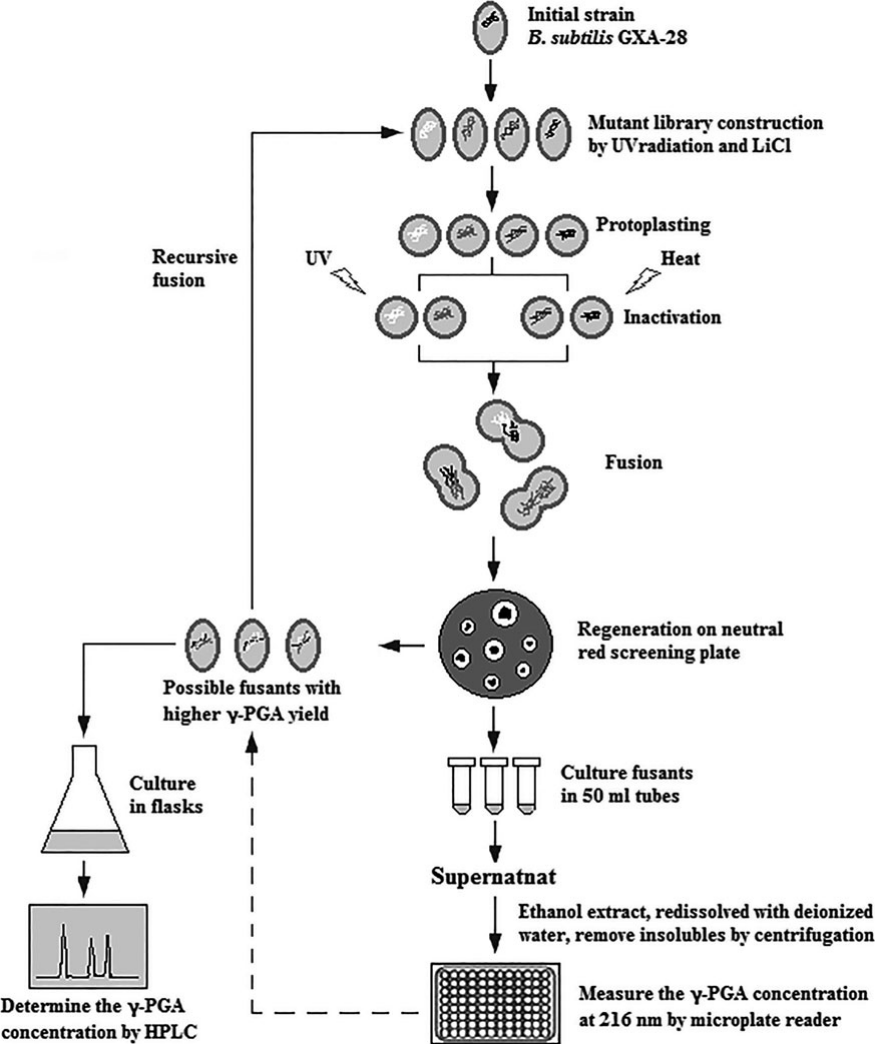
The procedure of genome shuffling and screening in *B. subtilis*. The process includes six steps, such as mutant library construction, protoplast preparation, inactivation, fusion, regeneration and fusants screening. Above steps were a round of genome shuffling, and three rounds were conducted in this study. Fusants screening was done through two steps: primary screening by neutral red plate and second screening by liquid culture. Finally, the target fusant was obtained by using UV or HPLC method to determine the γ‐PGA concentration in the fermentation broth.

The procedure of genome shuffling includes six steps, such as mutant library construction, protoplast preparation, inactivation, fusion, regeneration and screening of fusants (Fig. 1). Thereinto, mutant library construction was the first step. In this study, *B. subtilis* GXA‐28 was mutagenized by UV irradiation or UV/LiCl.

**Figure 2:**
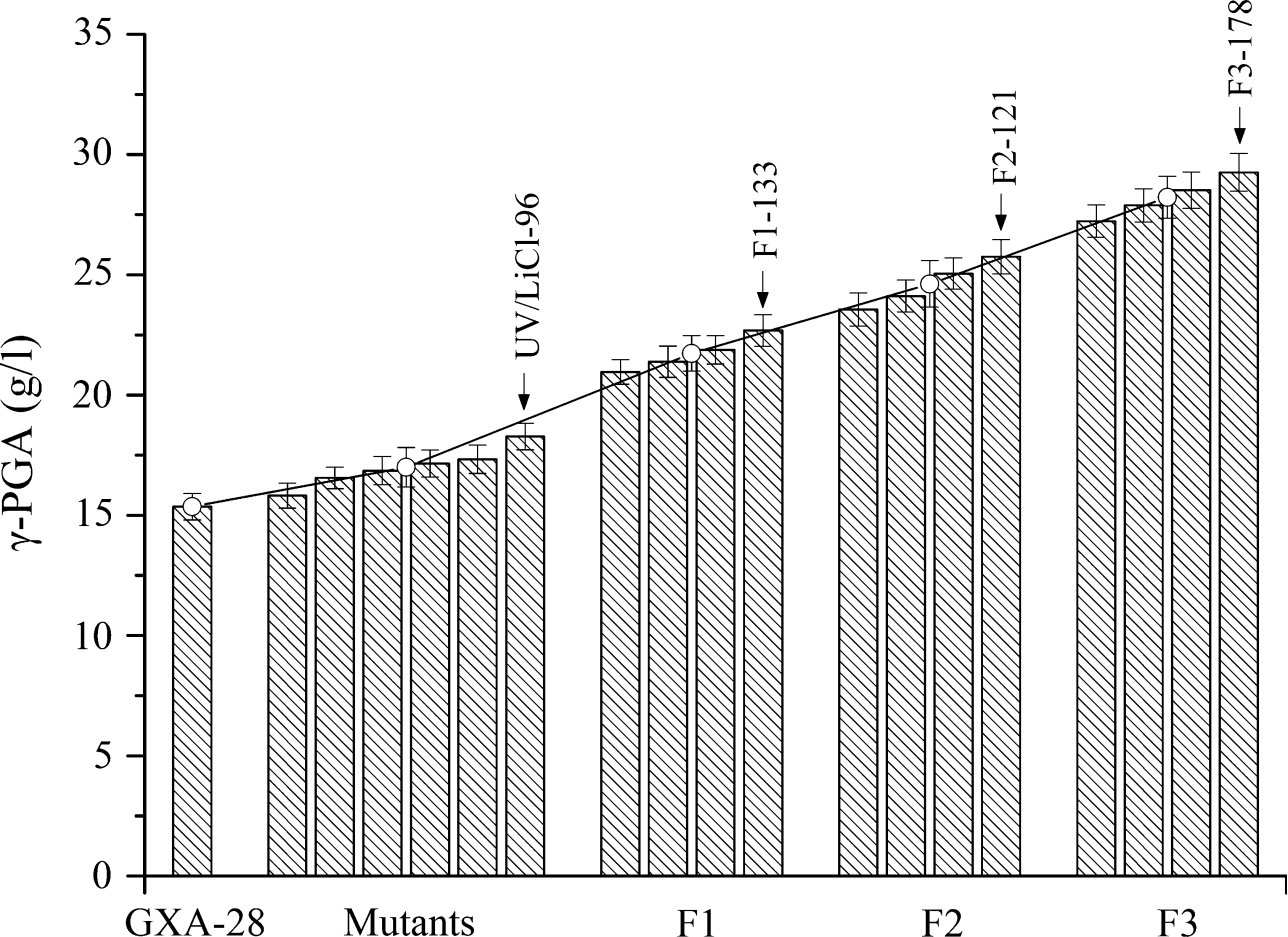
The γ‐PGA yield of parental strain (GXA‐28) and mutants (UV, UV/LiCl, F1, F2 and F3) in shake flask.

A lethal rate of 90% and a positive mutation rate of 13.3% were achieved in UV mutants. The positive mutation rate reached 19.7% in UV/LiCl mutants. The average of γ‐PGA yield of six mutants (the three UV and three UV/LiCl mutants) was 17.0 ± 0.6 g l^−1^ (range: 15.8 ± 0.5 to 18.3 ± 0.6 g l^−1^), which was 10.7% higher than the parental strain GXA‐28 (15.3 ± 0.5 g l^−1^) (Fig. 2). The yield remained stable after 20 subcultures, suggesting that UV radiation and UV/LiCl are efficient mutation method for GXA‐28.

### Genome shuffling

Mutants with improved phenotypes and diverse genetic sequences can be used for genome shuffling, a method to further increase natural evolution via the recursive homologous recombination. The procedure of genome shuffling and screening are displayed in Fig. 1.

Three hundred fusants were obtained after 1st round genome shuffling, among which, four strains (F1) with highest γ‐PGA yield were obtained and the yield was confirmed by shake flask fermentation. The average γ‐PGA yield of the four F1 (21.7 ± 0.6 g l^−1^) was 27.8% higher than that of the UV and UV/LiCl mutants and 41.8% higher than that of the parental strain GXA‐28. F1 were used for the 2nd round genome shuffling and 300 fusants were obtained. Four strains with the highest γ‐PGA yield were obtained (F2, average of 24.6 ± 0.7 g l^−1^) and used for the 3rd round. Four strains were then obtained in the 3rd round (F3, average of 28.2 ± 0.7 g l^−1^). The UV/LiCl strains (UV/LiCl‐96, 18.3 ± 0.6 g l^−1^) and three strains with the highest yield at each round (F1‐133, 22.7 ± 0.7 g l^−1^; F2‐121, 25.8 ± 0.7 g l^−1^; and F3‐178, 29.3 ± 0.8 g l^−1^) were collected for analysis. γ‐PGA yield of F3‐178 remained stable after 20 subcultures, indicating that genome shuffling technology could be applied in *B. subtilis* to improve γ‐PGA production.

### Morphological characteristics of parental (GXA‐28) and shuffled strain (F3‐178)

**Figure 3:**
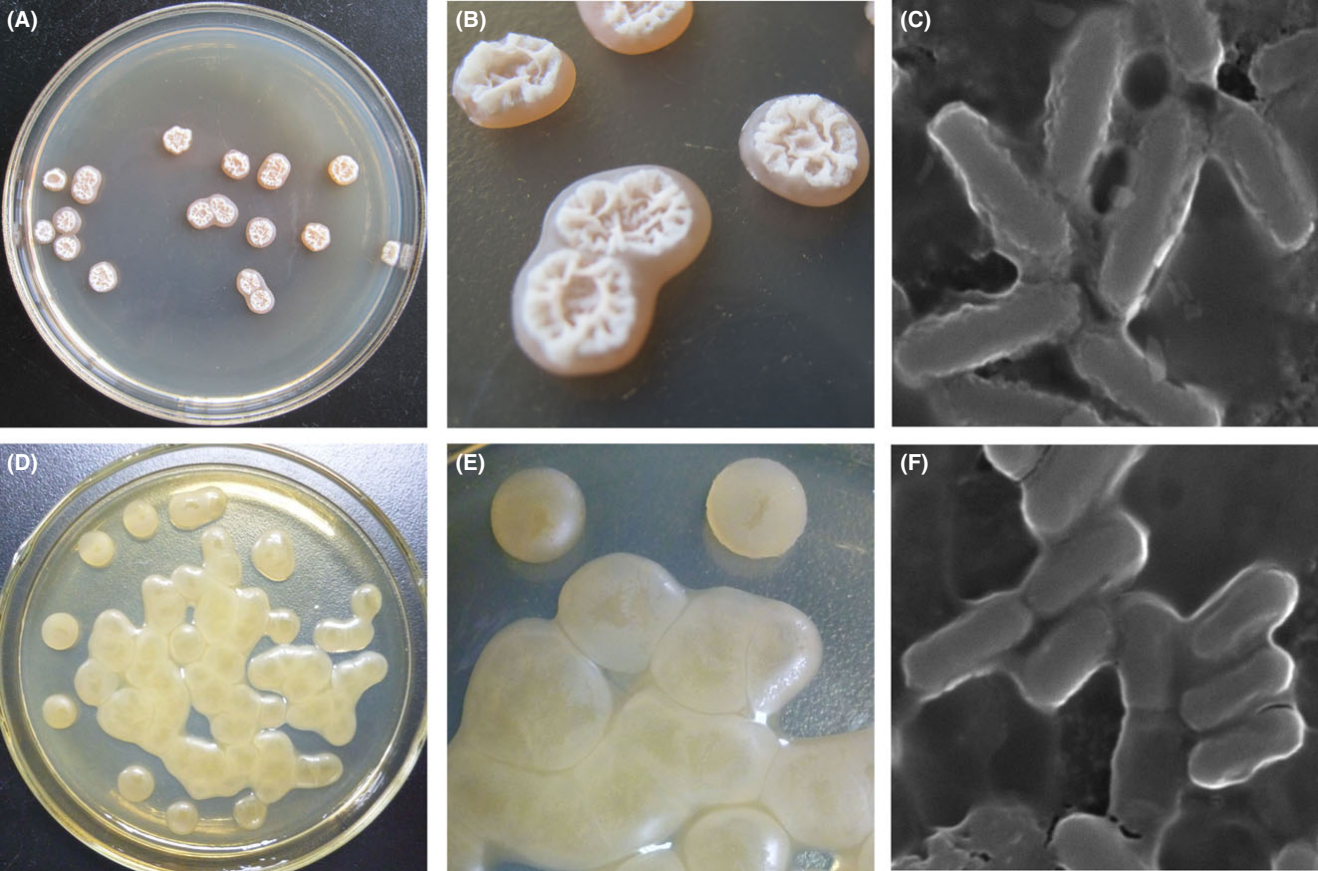
Characteristic of parental (GXA‐28) and mutants (UV, UV/LiCl, F1, F2 and F3) on agar, which were observed by Olympus E330 camera (Olympus) and Hitachi S‐3400N scanning electron microscope (Hitachi Science Systems, Japan). (A) GXA‐28; (B) enlarged view of GXA‐28; (C) cells of GXA‐28 (×10,000); (D) F3‐178; (E) enlarged view of F3‐178; (F) cells of F3‐178 (×10,000).

Because random breeding technology usually leads to changes in morphological traits, we compared the morphological characteristics of the parental strain (GXA‐28) and the shuffled strain (F3‐178) on agar containing glutamate after 24‐h incubation (Fig. 3). GXA‐28 on agar was round (0.6 cm in diameter), surrounded with slime and covered with milk‐white folding mycoderm (Fig. 3A and B). F3‐178 was yellow, droplet‐shape (0.8 cm in diameter) and covered with a layer of thin pellicle that wrapped a mass of viscous liquid (Fig. 3D and E). The formation of bacterial lawn, a field or mat of bacteria colonies, was faster in F3‐178. GXA‐28 cells were rod‐shaped (3.0 μm in length), surrounded by mucous envelope and connected with each other (Fig. 3C). F3‐178 colonies were also surrounded by thick mucous envelope, but the cell length was shorter (2.0 μm in length) (Fig. 3F). These results demonstrated that the morphological characteristics of F3‐178 were not identical to the parental strain.

### γ‐PGA production in fermenter

**Figure 4:**
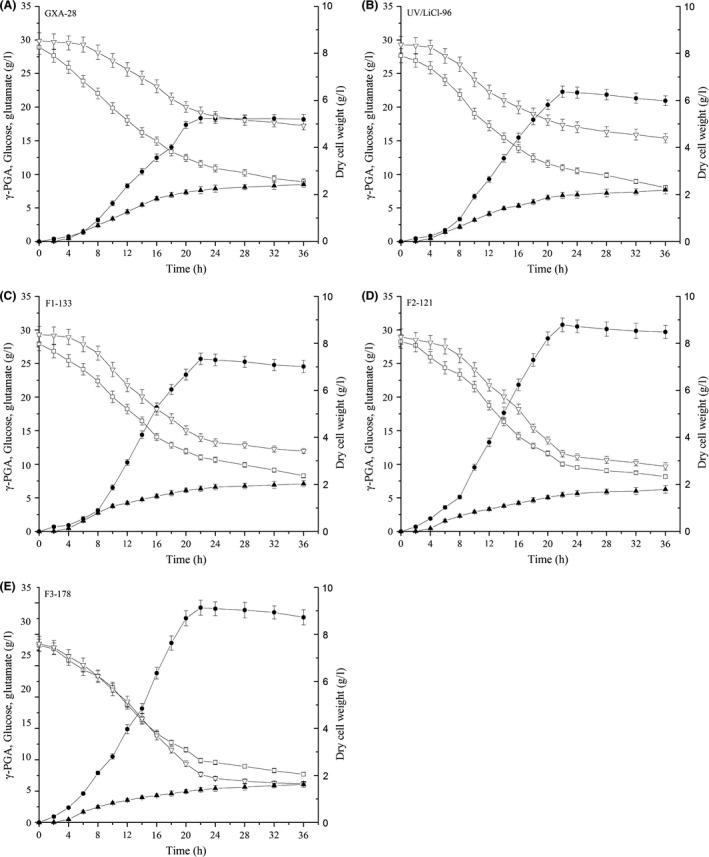
Time‐courses of γ‐PGA production in 3.6 l fermenter. (A) GXA‐28, (B) UV/LiCl‐96, (C) F1‐133, (D) F2‐121, (E) F3‐178. γ‐PGA concentration (g l^−1^, ●); dry cell weight (g l^−1^, ▲); glucose concentration (g l^−1^, □); glutamate concentration (g l^−1^, ▽).

**Figure 5:**
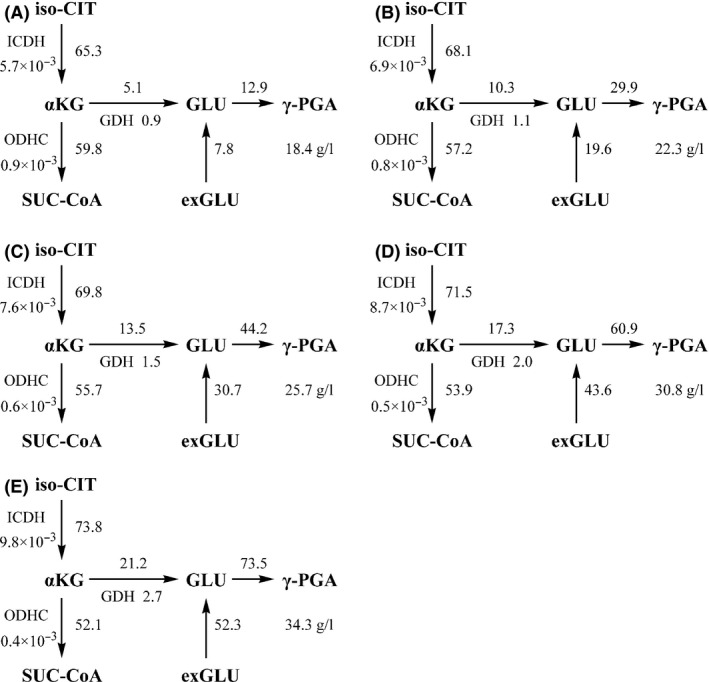
Carbon flux distribution and enzyme activities at α‐oxoglutarate and γ‐PGA synthesis branch point. (A) GXA‐28; (B) UV/LiCl‐96; (C) F1‐133; (D) F2‐121; (E) F3‐178. The metabolic fluxes were given in units of mmol g^−1^ DWC ^−1^ h^−1^. The unit of enzyme activity was U mg^−1^ protein. Abbreviations: iso‐CIT, isocitrate; αKG, α‐oxoglutarate; SUC‐CoA, succinyl coenzyme A; GLU, glutamate; exGLU, exogenous glutamate; γ‐PGA, poly‐γ‐glutamic acid; ICDH, isocitrate dehydrogenase; ODHC, α‐oxoglutarate dehydrogenase; GDH, glutamate dehydrogenase.

We compared the fermentation properties of the parental strain (GXA‐28) and mutants (UV/LiCl‐96, F1‐133, F2‐121 and F3‐178) (Fig. 4). The highest γ‐PGA yield reached at 22 h in all strains. Compared with GXA‐28, the yield was 1.9‐fold higher in F3‐178 (34.3 ± 1.2 g l^−1^), but the DCW (dry cell weight) in F3‐178 (1.4 ± 0.1 g l^−1^) was 36.4% less than GXA‐28. The glutamate in medium of F3‐178 was significantly lower than that in GXA‐28 (7.7 ± 0.3 g l^−1^ versus 19.2 ± 0.8 g l^−1^). In F3‐178, the total amount of glutamate (in γ‐PGA and medium) was more than the initial added amount. It is possible that the substrate of γ‐PGA came from two parts: one was from extracellular glutamate which exists in the medium by artificial added, and others derived from intracellular glutamate which obtained via glycolysis and tricarboxylic acid cycle by glucose (Ogunleye *et al*., 2015). Although there were obviously differences in the γ‐PGA yield and DCW between parental strain and mutants, the residual glucose was comparable in all strains (8 g l^−1^). Furthermore, the carbon flux from glucose to glutamate increased 4.1‐fold in F3‐178 (21.2 mmol DCW^−1^ h^−1^) compared with that in GXA‐28 (5.1 mmol DCW^−1^ h^−1^) (Fig. 5), while the carbon flux from glucose to biomass formation decreased 1.7‐fold in F3‐178 (2.5 mmol DCW^−1^ h^−1^) compared with that in GXA‐28 (4.2 mmol DCW^−1^ h^−1^) (Table 1). This result suggested that more glucose was used to support the γ‐PGA production in F3‐178.

**Table 1:** Metabolic fluxes in parental and mutants

Metabolic reactions	Metabolic fluxes (mmol g^−1^ (DCW)^−1^ h^−1^)				
	GXA‐28	UV/LiCl‐96	F1‐133	F2‐121	F3‐178
r (FADH_2_ ↔ ATP)^a^	52.7	55.3	57.9	59.6	60.5
r (NADPH ↔ ATP)^b^	93.5	100.7	110.2	117.8	122.6
r (NADH ↔ ATP)^c^	252.6	285.8	319.5	367.1	395.2
r (Biomass formation)^d^	4.2	3.7	3.4	2.8	2.5

**a,b,c**. Metabolic reactions of energy metabolism and biomass formation of *B. subtilis* was based on a previous metabolism model (Zeng *et al*., 2014). (a): 1 FADH_2_+ 0.5 O_2_ ↔0.8667 ATP + 1 FAD + 1 H_2_O; (b): 1 NADPH + 0.5 O_2_ ↔1.3 ATP + 1 NADP + 1 H_2_O. (c): 1 NADH + 0.5 O_2_ ↔1.3 ATP + 1 NAD + 1 H_2_O.

**d**. The biomass composition of *B. subtilis* was based on the published data (Sauer *et al*., 1996). (d): 0.000154G6P + 0.00019 F6P + 0.000194 GAP + 0.001353 PG3 + 0.000711 PEP + 0.00306 PYR + 0.002132 AcCoA + 0.016274 ATP + 0.000816 Ru5P + 0.016058 NADPH + 0.000308 E4P + 0.001071 αKG + 0.001923 OAA→Biomass + 0.003488 NADH + 0.002873 CO_2_.

Interestingly, a certain amount of glutamate could be detected in medium even after 36‐h fermentation in all strains, such as, 17.3 ± 0.6 g l^−1^ for GXA‐28 and 6.2 ± 0.3 g l^−1^ for F3‐178. On the other hand, the strains could not produce γ‐PGA in the absence of glutamate (Zeng *et al*., 2014). It indicated that exogenous glutamate was essential for the production of γ‐PGA. This phenomenon is in line with a previous observation in *B. licheniformis* ATCC 9945A (Cromwick and Gross, 1995). Cromwick *et al*. suggested that citrate is a precursor substrate for γ‐PGA production, and the exogenous glutamate is an inducer. Our finding further supports that glutamate serves as an inducer in γ‐PGA production in GXA‐28 and mutants.

For polymers production, such as pullulan and ε‐poly‐l‐lysine (ε‐PL), mutants with 1–2 times higher yield is more suitable for industrial application, which is different from the strains used for production of enzymes or antibiotics. For example, the yield of pullulan in *Aureobasidium pullulans* F3‐2 and ε‐PL in *Streptomyces graminearus* F3‐4 is approximately 80% higher than their parental strains (Kang *et al*., 2011; Li *et al*., 2011). The authors explained that when polymer with relative high molecular weight (> 10^6^ Da for polysaccharide and γ‐PGA) is higher than 20 g l^−1^, the viscosity of fermentation liquid could increase significantly, resulting in decreased yield. Therefore, F3‐178 with 90% higher yield (reached 34.3 ± 1.2 g l^−1^) than GXA‐28 could be used for industrial production of γ‐PGA.

### Metabolic analysis of γ‐PGA biosynthesis

The metabolic flux and enzymatic activities of key metabolic branch, including energy metabolism, biomass formation and α‐oxoglutarate branch were measured to investigate the effect of UV/LiCl mutation and genome shuffling on γ‐PGA biosynthesis. Table 1 shows higher ATP in F3‐178 than other strains, which agrees with a previous report that ATP is required for γ‐PGA synthesis (Ashiuchi, 2013). The carbon distribution to biomass in F3‐178 was lower than in other strains, which may be because the activity energy mainly used for γ‐PGA synthesis.

The α‐oxoglutarate is a crucial part of the γ‐PGA biosynthesis pathway, where carbon flux is divided into succinyl‐CoA and intracellular glutamate (Yao *et al*., 2010). Compared with GXA‐28, carbon flux from isocitrate to α‐oxoglutarate increased, but carbon flux from α‐oxoglutarate to the succinyl‐CoA decreased in other strains (Fig. 5). Correspondingly, the enzyme activity of isocitrate dehydrogenase (ICDH) was increased from (5.7 ± 0.4) × 10^−3^ U mg^−1^ protein in GXA‐28 to (9.8 ± 0.7) × 10^−3^ U mg^−1^ protein in F3‐178, but the enzyme activity of α‐oxoglutarate dehydrogenase complex (ODHC) was decreased from (0.9 ± 0.06) × 10^−3^ U mg^−1^ protein in GXA‐28 to (0.4 ± 0.03) × 10^−3^ U mg^−1^ protein in F3‐178. In addition, the carbon flux from α‐oxoglutarate to glutamate (21.2 mmol DCW^−1^ h^−1^) increased 4.1‐fold in F3‐178 than in GXA‐28. Furthermore, the activity of glutamate dehydrogenase (GDH) was 3.0‐fold higher in F3‐178 (2.7 ± 0.2 U mg^−1^ protein) than in GXA‐28 (0.9 ± 0.05 U mg^−1^ protein). The extracellular glutamate uptake rate was 7.8 mmol DCW^−1^ h^−1^ in GXA‐28, and increased to 52.3 mmol DCW^−1^ h^−1^ in F3‐178. Taken together, the higher γ‐PGA yield in F3‐178 could be attributed to increased intracellular flux and uptake of extracellular glutamate.

We examined the mRNA level of *pgs*B, a gene of the γ‐PGA synthase complex that is responsible for catalysing glutamate to γ‐PGA (Kimura *et al*., 2009; Ashiuchi, 2013) (Table 2). The mRNA level of *pgs*B in F3‐178 was 18.8‐fold higher than in GXA‐28, supporting the key role of *pgs*B in γ‐PGA production in F3‐178. More studies are needed to examine the level of other genes participated in γ‐PGA production, such as *pgs*E and *deg*Q (Do *et al*., 2011; Yamashiro *et al*., 2011).

**Table 2:** mRNA level of *pgs*B in parental and mutants from RT‐PCR

Strains	CT_*pgs*B_	CT_16s rDNA_	Delta CT^a^	Delta‐delta CT	Relative concentration
GXA‐28	26.5 ± 0.4	16.0 ± 0.2	10.4 ± 0.3	0	1.0
UV/LiCl‐96	25.5 ± 0.3	16.4 ± 0.1	9.1 ± 0.3	−1.3 ± 0.1^b^	2.5 ± 0.1
F1‐133	24.9 ± 0.3	16.6 ± 0.4	8.3 ± 0.3	−2.1 ± 0.1^c^	4.4 ± 0.2
F2‐121	23.6 ± 0.4	16.6 ± 0.2	7.0 ± 0.4	−3.4 ± 0.1^d^	10.9 ± 0.5
F3‐178	22.7 ± 0.4	16.5 ± 0.3	6.2 ± 0.3	−4.2 ± 0.1^e^	18.8 ± 1.6

a CT_*pgs*B_ ‐ CT_16s rDNA_.

b delta CT_UV/LiCl‐96_ ‐ delta CT_GXA‐28_.

c delta CT_F1‐133_ ‐ deltaCT_GXA‐28_.

d delta CT_F2‐121_ ‐ deltaCT_GXA‐28_.

e delta CT_F3‐178_ ‐ deltaCT_GXA‐28_.

## Conclusions

In this study, we developed a mutant, *B. subtilis* F3‐178, using genome shuffling technology. The γ‐PGA yield of F3‐178 was significantly higher than the parental strain GXA‐28 in 3.7 l fermenter, and it is satisfactory for industrial production of γ‐PGA. The higher yield could be attributed to increased intracellular flux and uptake of extracellular glutamate and might be related to the overexpression of genes involved in γ‐PGA production, such as *pgs*B. This study demonstrated that genome shuffling can be used for rapid improvement of γ‐PGA strains. Then the possible mechanism for the improved phenotype was also explored at the metabolic and transcriptional levels.

## Experimental procedures

### Bacterial strain and culture conditions

*B. subtilis* GXA‐28 strain from China Center for Type Culture Collection (CCTCC M 2012347) was used in our study (Zeng *et al*., 2013). The seed medium is composed of glucose 10.0 g l^−1^, yeast extract 5.0 g l^−1^, L‐glutamate 5.0 g l^−1^, KH_2_PO_4_ 0.5 g l^−1^, K_2_HPO_4_·3H_2_O 0.5 g l^−1^ and MgSO_4_·7H_2_O 0.1 g l^−1^. 0.006% (w/v) neutral red (dye) and agar (1.5%, w/v) were added into seed medium to prepare screening plate (Zeng *et al*., 2013). In fermentation medium, glucose, yeast extract and L‐glutamate were adjusted to 30.0, 2.5 and 30.0 g l^−1^ respectively. The protoplast stable liquid, SMM, sucrose, MgCl_2_·6H_2_O, maleic acid, composed of sucrose 0.5 mol l^−1^, MgCl_2_·6H_2_O 0.02 mol l^−1^ and maleic acid 0.02 mol l^−1^ (pH 6.5) was sterilized at 115°C for 30 min (Zhao *et al*., 2012). The components of regeneration medium (RM) were the same as seed medium, but prepared in SMM as solvent instead of distilled water. Agar (1.2%, w/v) was added to RM to prepare RM plates. Lysozyme (Sigma‐Aldrich, St. Louis, MO, USA) was added into SMM (10.0 mg ml^−1^), sterilized by filtration through a 0.22 μm membrane and stored at −20°C. PEG 6000 (35%, w/v) and CaCl_2_ (10 mM) were added into SMM as fusogen medium.

### Mutagenesis

GXA‐28 cells were inoculated into 30 ml seed medium and aerobically cultured at 45°C for 12 h with shaking at 200 r.p.m. The log‐phase cells were collected by centrifugation at 4°C and 8,000 *g* for 5 min, washed twice with sterilized deionized water and re‐suspended in 10 ml Tris–HCl buffer (25 mM, pH 6.0). Some aseptic plates containing 5 ml suspensions were exposed to UV radiation (15 W) at a vertical distance of 30 cm for 60 s to generate UV mutants. Some other aseptic plates containing 5 ml suspensions and lithium chloride (LiCl, 0.6%, w/v) were exposed to UV radiation (15 W) at a vertical distance of 30 cm for 60 s to generate UV/LiCl mutants. The surviving cells was diluted, spread onto the screening plates and incubated in the dark at 45°C. Five hundred UV or UV/LiCl mutants were selected to grow in 50 ml tubes with 5 ml fermentation medium. Ten UV and 10 UV/LiCl mutants with the highest yield of γ‐PGA were chosen for secondary screening by shake flask fermentation. Finally, three UV and three UV/LiCl mutants with the highest γ‐PGA yield were selected for protoplast fusion experiments.

### Protoplast preparation

Protoplasts were prepared as described previously with modifications (Zhao *et al*., 2012). Strains were harvested from seed medium by centrifugation, washed and suspended in SMM to an optical density at 660 nm of 2.0 using UV‐mini 1240 spectrophotomer (Shimadzu, Kyoto, Japan). The suspension was treated by 1.0 mg ml^−1^ lysozyme at 37°C for 20 min and the appearance of protoplasts (round forms) was monitored under light microscope (Olympus CX41; Olympus, Tokyo, Japan). After centrifuge, protoplasts were suspended in SMM buffer and protoplast formation ratio (%) was calculated as: [(A−B)/A] × 100%, where A and B represent colony‐forming units (cfu) on seed medium plate before and after protoplasts preparation, individually.

### Protoplast inactivation

The protoplasts (1.0 × 10^7^ cells ml^−1^) was inactivated by heat treatment (100°C water bath for 20 min) or UV irradiation (15 W at a vertical distance of 30 cm for 120 s) (Shi *et al*., 2014). After treatment, protoplasts were maintained in the dark for 2 h to avoid photo‐reactivation repair. The protoplast inactivation ratio (%) was calculated as: [1−(A−B)/(C−D)] × 100%, where A and B represent cfu on RM plate and seed medium plate after inactivation; C and D represent cfu on RM plate and seed medium plate before inactivation.

### Protoplast fusion and regeneration

The protoplasts from different mutants (1.0 × 10^7^ cells ml^−1^) were mixed in equal proportion. Half of the mixture was inactivated with UV, and half was inactivated with heat treatment. The inactivated solution was mixed again, centrifuged at 8,000 *g* for 5 min, re‐suspended in 10 ml fusogen medium and incubated at 37°C for 10 min. After centrifuge, the fused protoplast was spread on RM plate after serial dilutions and cultured at 37°C for 36 h.

### Screening of mutants and fusants

After grown on screening plates, colonies with the large concentric colour halo were inoculated in 50 ml tubes containing 5 ml fermentation medium. γ‐PGA yield was measured by ultraviolet spectrophotometry (UV) or high efficiency liquid chromatography (HPLC) assay (Zeng *et al*., 2012). The molecular weight of γ‐PGA was determined by gel permeation chromatography (Zeng *et al*., 2013).

### Genome shuffling

Above steps, composed of mutagenesis, protoplasts preparation, inactivation, fusion, regeneration and fusants screening, were a round of genome shuffling. The selected colonies, first generation (F1), were used as the parental strain for subsequent round of genome shuffling. The target strain was obtained after three rounds of genome shuffling (Fig. 2). After 20 generations, the strains with comparable γ‐PGA yield as the first generation by 250 ml flask fermentation were considered to be genetically stable.

### Culture conditions in shake flask and fermenter

Fermentation in 250 ml flask and 3.6 l fermenter (INFORS HT, Basel, Switzerland) was performed as our previous study (Zeng *et al*., 2014).

### Metabolic flux analysis

The metabolic model for γ‐PGA biosynthesis in *B. subtilis* GXA‐28 was constructed based on method developed in our lab (Zeng *et al*., 2014). The metabolic reaction matrixes could be solved by the least squares approach (Matlab 7.0; MathWorks, Neddick, MA, USA). The specific rates of glucose uptake, glutamate uptake, O_2_ uptake, γ‐PGA synthesis, cell growth and CO_2_ evolution were measured to calculate metabolic flux distributions when strains were cultured for 20 h, which was at the late‐exponential stage (Table 3). All the flux distributions were normalized by the glucose uptake rate on a basis of 100 mmol g^−1^ (DCW)^−1^ h^−1^. The concentrations of glucose and L‐glutamate were measured by a biosensor (SBA‐40D; Shandong Academy of Sciences, Jinan, China). O_2_ and CO_2_ concentrations in the exhaust gas were analysed by gas analyser (LKM2000A; Lokas, Daejeon, Korea). DO and pH values were measured online by electrodes of the fermenter.

**Table 3:** The physiological parameters of parental and mutants at the late‐exponential stage in 20 h

Strains	Cell growth rate (×10^−2^, h^−1^)	Glucose uptake rate (×10^−1^, h^−1^)	Glutamate uptake rate (×10^−1^, h^−1^)	γ‐PGA formation rate (×10^−1^, h^−1^)	O_2_ uptake rate (mmol g^−1^ (DCW)^−1^ h^−1^)	CO_2_ evolution rate (mmol g^−1^ (DCW)^−1^ h^−1^)
GXA‐28	2.1	1.7	2.1	3.8	19.1	12.7
UV/LiCl‐96	1.6	2.2	3.0	5.4	17.3	9.6
F1‐133	2.0	3.1	4.3	7.0	16.6	8.8
F2‐121	2.4	4.9	6.1	10.1	13.7	6.7
F3‐178	2.3	5.8	8.2	13.0	11.5	5.9

### Enzyme activity assays

Strains grown in batch fermentation for 20 h were harvested by centrifugation, and cell extracts were prepared for determination of ICDH, GDH and ODHC activities. Enzyme activities were determined by appearance or disappearance of NADH or NADPH (*ε* = 6.22/(mmol l^−1^) cm^−1^) at 340 nm in 3 ml reaction mixture (Zeng *et al*., 2014). One unit of activity was defined as the amount of enzyme catalysing 1 μmol of NADH or NADPH per min. Protein concentration was determined by BCA method (Smith *et al*., 1985).

### RT‐PCR

Strains grown in batch fermentation for 20 h were harvested by centrifugation, and RNA extraction and reverse transcriptase reaction were performed using the EZ‐10 DNAaway RNA Mini Preps Kit (B618133; Bio Basic Inc., Markham, ON, Canada) and the Revert Aid First Strand cDNA Synthesis Kit (K1621; Thermo Scientific, Waltham, MA, USA) respectively. RT‐PCR was performed using 2× SG Fast qPCR Master Mix kit (B639271, SYBR Green; Bio Basic Inc., Markham, ON, Canada) on Roche Light Cycle 480 to analyses *pgs*B (GeneBank: KP178960, the target gene) and 16s rDNA (GeneBank: JN815234, the reference gene). The primers were designed by the Primer Premier 5.0 software (PREMIER Biosoft, Palo Alto, CA, USA): forward 5′‐GAGCGAGCCTGGGCACTT‐3′ and reverse 5′‐CGGTCATCGGTCGGGTAAC‐3′ (*pgs*B); forward 5′‐TGCCGCAATGGA CGAAAG‐3′ and reverse 5′‐TGTAAGGTGCCGCCCTATTC‐3′ (16s rDNA). Relative gene quantification was performed using the comparative 2^−ΔΔCT^ method and normalized to 16s rDNA (Livak and Schmittgen, 2001).

## Conflict of Interest

None declared.
